# Supplementary material for: Regulation of Female Folliculogenesis by Tsp1a in Nile Tilapia (Oreochromis niloticus)
Source: Int J Mol Sci. 2020 Aug 16;21(16):5893. doi: 10.3390/ijms21165893 (PMC7460569; doi:10.3390/ijms21165893)
Supplement: Supplementary file 1 [file ijms-21-05893-s001.pdf]

**Figure S1**

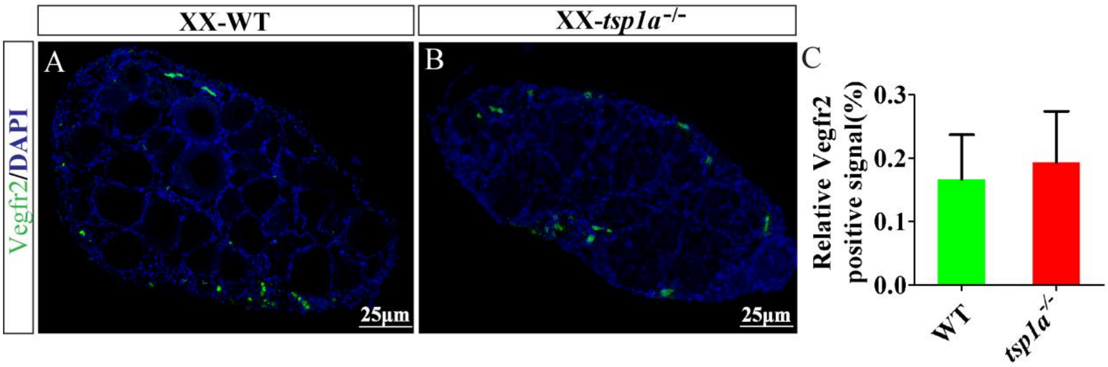


**Figure S1.** Blood vessel density in ovaries from *tsp1a*$^{-/-}$ and WT fish at 120dah. **(A-B)** Immunofluorescence analysis of Vegfr2. Green fluorescence represents the positive signal. (**C**) Statistical analysis of the positive signals ($n$ = 5, and five sections per sample were counted). Difference between the mutants and WT were tested by two-tailed unpaired Student's t-test. Results were presented as the mean ± SD in C. WT, wild type.
